# Morphological Variations of Leading-Edge Serrations in Owls (*Strigiformes*)

**DOI:** 10.1371/journal.pone.0149236

**Published:** 2016-03-02

**Authors:** Matthias Weger, Hermann Wagner

**Affiliations:** Institute of Zoology, RWTH Aachen University, Aachen, Germany; University of Lethbridge, CANADA

## Abstract

**Background:**

Owls have developed serrations, comb-like structures, along the leading edge of their wings. Serrations were investigated from a morphological and a mechanical point of view, but were not yet quantitatively compared for different species. Such a comparative investigation of serrations from species of different sizes and activity patterns may provide new information about the function of the serrations.

**Results:**

Serrations on complete wings and on tenth primary remiges of seven owl species were investigated. Small, middle-sized, and large owl species were investigated as well as species being more active during the day and owls being more active during the night. Serrations occurred at the outer parts of the wings, predominantly at tenth primary remiges, but also on further wing feathers in most species. Serration tips were oriented away from the feather rachis so that they faced into the air stream during flight. The serrations of nocturnal owl species were higher developed as demonstrated by a larger inclination angle (the angle between the base of the barb and the rachis), a larger tip displacement angle (the angle between the tip of the serration and the base of the serration) and a longer length. Putting the measured data into a clustering algorithm yielded dendrograms that suggested a strong influence of activity pattern, but only a weak influence of size on the development of the serrations.

**Conclusions:**

Serrations are supposed to be involved in noise reduction during flight and also depend on the aerodynamic properties that in turn depend on body size. Since especially nocturnal owls have to rely on hearing during prey capture, the more pronounced serrations of nocturnal species lend further support to the notion that serrations have an important function in noise reduction. The differences in shape of the serrations investigated indicate that a silent flight requires well-developed serrations.

## Introduction

**Fig 1:**
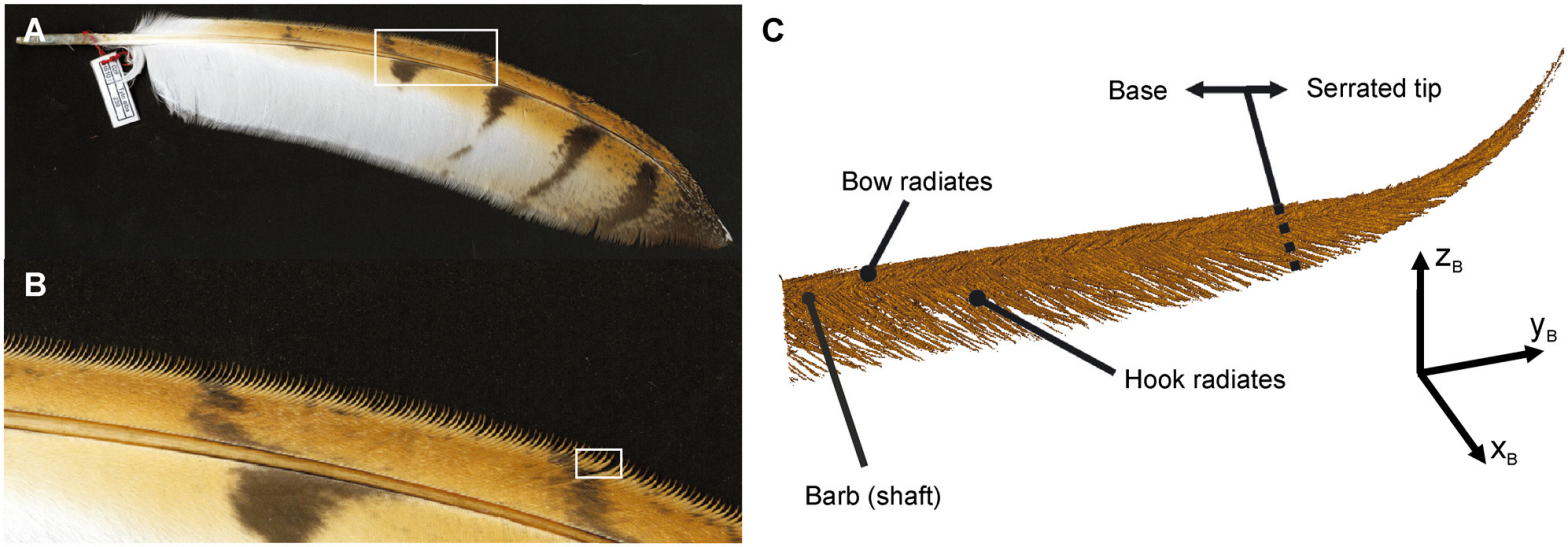
Serrations of *T*. *furcata pratincola* in different magnifications. (A) Photograph of a 10^th^ primary showing serrations along the outer vane. (B) Close up as indicated by the white rectangle in (A). Now the bending of the serrations is clearly visible. (C) Three-dimensional reconstruction of a serration. The serration was taken from the region marked by the white rectangle in (B). The barb shaft and the bow and hook radiates can be seen. The serration is divided into an unbent base and a serrated tip. The coordinate system, with the x-axis in the direction of the unbent barb shaft, the y-axis in the plane of the radiates with the positive direction towards the tips of the hook radiates, and the z-axis directed upwards, will be used in the further descriptions (adapted from [6]).

Owls (Strigiformes) have developed a silent flight. These birds have acquired several specializations on their wings and feathers. These structures have been implied in the reduction of noise production during flight [1,2]. One conspicuous adaptation is the presence of serrations at the leading edge of the wing [1,2] (Fig 1). The serrations are formed by barb endings. Many authors have noticed the existence of the serrations (e.g. [1,3–7]) and provided some information on the shape of leading-edge serrations and their variation with the position on a feather, yet to our best knowledge a precise definition of a serration has not been provided.

For example, Bachmann and Wagner [6] provided a detailed description of leading-edge serrations of 10th primary feathers of the American barn owl (*Tyto furcata pratincola*), and mentioned that the serrations are bent away from the rachis and upwards. Mascha [3] stated that serrated feather structures do not only occur on owl wings, but also on the wings of the kakapo (*Strigops habroptilus*) and frogmouths (*Podargus strigoides*). Serrations do not only occur on the 10^th^ primaries as in the barn owl, but also on other primaries [1]. Sick [4] proposed a classification of leading-edge serrations into two different categories, the “highly toothed” (Bubo type) and the “weakly toothed” (Surnia type) group, with the Surnia type serrations being less bent. However, Sick [4] did not provide further quantification of the morphology of the serrations of different species, and mentioned that it would be worthwhile to study serration shape in more detail. To our best knowledge, no one has followed up Sick’s work. What is also not well known is how the size, the shape, and the occurrence of the serrations varies between owl species [4,5]. Thus, the description of the serrations needs an improvement to allow a better prediction of their function. We here provide such an improved description.

**Fig 2:**
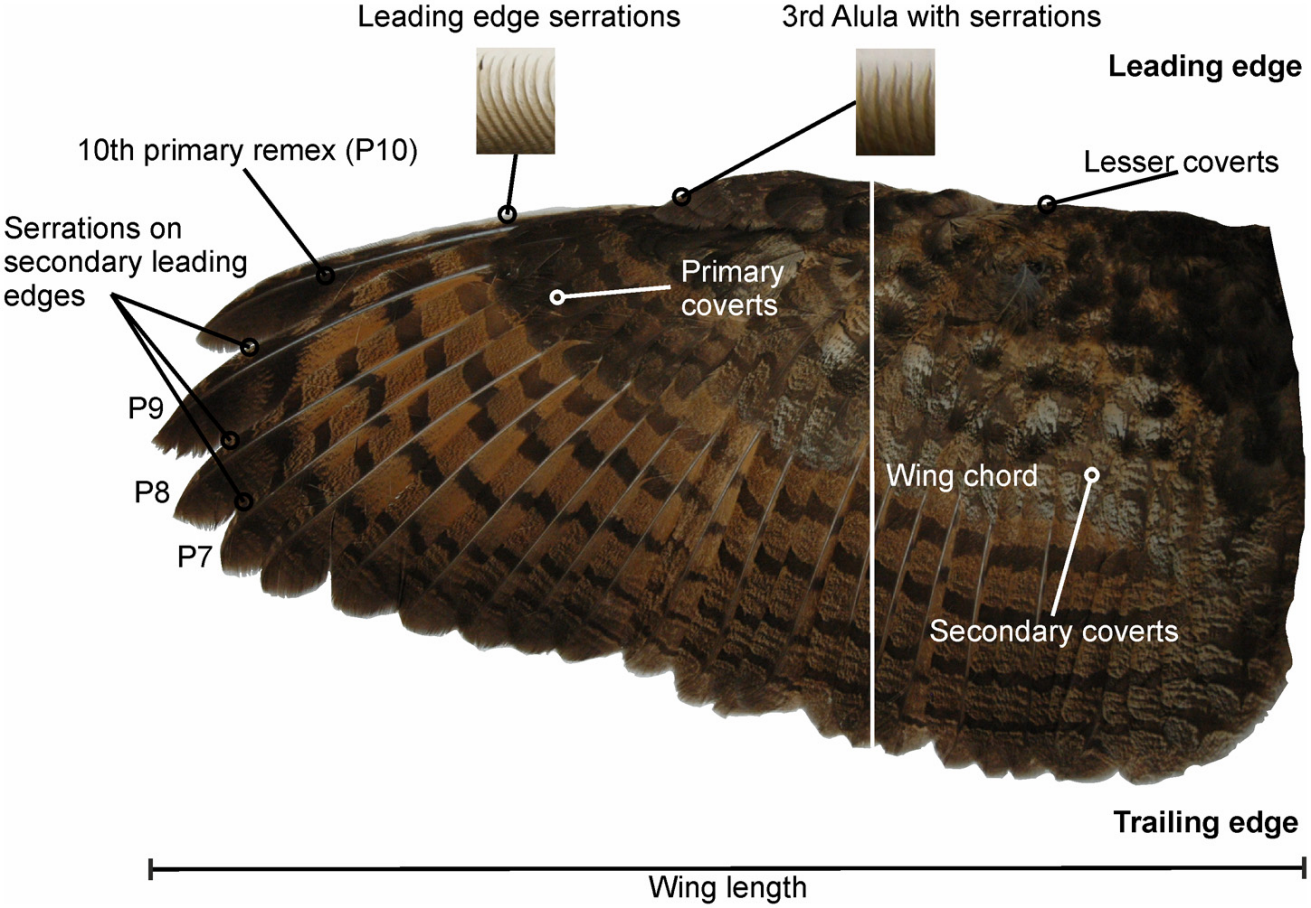
Wing of *B*. *bubo*. Leading-edge serrations occur on the 10^th^ primary remex and the 3^rd^ alula. Serrations occur also on the 9^th,^ 8^th^ and 7^th^ primary remiges.

Owls are an order of birds of prey with about 200 species [8]. Owl species vary in size over a huge range. The smallest owl is the pygmy owl (*Glaucidium passerinum*, weight: 42 g, length: 150–190 mm [9]). The largest owl is the eagle owl (*Bubo bubo*, weight: up to 4200 g, length: 580–710 mm [9] (see wing in Fig 2, see also S1 Table)). We reasoned that this range should be large enough to see an effect of size. Thus, our first hypothesis was that the size of an owl species might have an influence on the morphology of the serrations. Moreover, the different owl species occupy a huge variety of ecological niches, and may live in deserts, woods as well as in the tundra. Some of the owl species are strictly nocturnal, like the boreal owl (*Aegolius funereus*), while other owl species are more diurnal, like the little owl (*Athene noctua*). The more diurnal owls do not have such highly developed adaptations to hunting by listening as the nocturnal owls do [10–12]. Since a silent flight plays a less important role during hunting in diurnal owls, we further hypothesized that the development of serrations may depend on the activity pattern of an owl species with nocturnal owls have more developed serrations than diurnal owls.

In this work we first discriminate serrated leading-edges from non-serrated leading-edges and derive a more precise definition of what should be called a serration than available so far. Furthermore, we present data that quantitatively assesses the variation of the shapes of leading-edge serrations of owl species that differ in size and activity pattern. These data demonstrate that serrations occur only in owls, that nocturnal species have higher developed serrations than diurnal species, and that size plays a smaller role than the activity pattern for the development of the serrations.

## Materials and Methods

### Ethics Statement

Several of the species we investigated are endangered or protected or both. No animals died for the purpose of this study. All samples for this study were obtained in the following manner: 1) Wings and feathers of *T*. *furcata pratincola* were available from the barn-owl colony of the Institute of Biology II at RWTH Aachen University. This material was obtained under a permit of local authorities (Landespräsidium für Natur, Umwelt und Verbraucherschutz Nordrhein Westfalen, Recklinghausen, Germany (LANUV)), typically from animals that had been sacrificed at the end of other experiments. 2) Feathers of the pigeon (*Columba livia domestica*) were obtained from a local breeder und a permit issued by local authorities (RWTH Aachen University, permit 1764A4). 3) All further samples analyzed in this study were obtained from third-parties. The authors had no involvement with the original collection of these samples. The material was obtained from different organizations under a permit from the Städteregion Aachen, Germany, from public institutions that received their samples and hold their collections under federal permits. The Zoological Research Museum Alexander König (ZFMK, Bonn, Germany) kindly provided samples of *the short-eared owl (Asio flammeus)*, *A*. *funereus*, *A*. *noctua*, the long-eared owl (*Asio otus*), *B*. *bubo*, the snowy owl (*Bubo scandiacus)*, the Eurasian skylark (*Alauda arvensis)*, the black-backed gull (*Larus fuscus*), the shrike (*Lanius spec*.), *P*. *strigoides* and *S*. *habroptilus*. Wageningen University and Research Centre (Wageningen, Netherlands) provided samples of *A*. *flammeus*, *A*. *noctua*, *A*. *otus* and *B*. *scandiacus*. The Naturhistorisches Museum (Basel, Switzerland) made available complete wings of *A*. *funereus*.

### Species investigated

The wings and primary feathers of seven owl species were investigated to compare the occurrence and shape of serrations (Table 1). The species were chosen to match the criteria for different sizes and different activity patterns (see S1 Table). We selected two large owl species (body weight higher than 1000 g: *B*. *bubo*, *B*. *scandiacus*), 3 middle-sized owl species (body weight between 300 and 1000 g: *T*. *furcata pratincola*, *A*. *otus*, *A*. *flammeus*), and two small owl species (below 300 g body weight: *A*. *funereus*, *A*. *noctua*) for the analysis (see S1 Table). When selecting the species, we noticed that size and phylogeny go in parallel ([13], see S1 Fig). Thus, size and phylogeny cannot easily be separated, meaning that size is not a good discrimination criterion for our primary goals. Nevertheless, body size might serve as a control criterion to see whether results with the second criterion are meaningful. The second criterion was lifestyle. We selected the species such that species with the same lifestyle were scattered throughout the phylogenetic tree ([9], see also S1 Fig). Thus, the large diurnal *B*. *scandiacus* is more closely related to the large nocturnal *B*. *bubo* than to the middle-sized diurnal *A*. *flammeus* or the small diurnal *A*. *noctua* (S1 Fig). The same holds for the other species as well (S1 Fig). The only exception is the nocturnal middle-sized *T*. *furcata pratincola* that is an outgroup to all other six species. We included *A*. *otus* as a nocturnal middle-sized owl in line with our main selection criteria, but also included *T*. *furcata pratincola*, because much is known about the biology of the species. All in all the owl species chosen represented examples for all possible combinations of size and activity pattern.

**Table 1:** Distribution of leading-edge serrations on different wing feathers of all owl species investigated.

Owl species/size	P10	P9	P8	P7	Alula3	gpc10
Large						
*B*. *bubo*	+	+	+	+	+	+
*B*. *scandiacus*	+	-	-	-	-	-
Middle sized						
*T*. *furcata*	+	-	-	-	-	+
*A*. *otus*	+	+	-	-	-	-
*A*. *flammeus*	+	+	-	-	-	-
Small						
*A*. *funereus*	+	+	+	-	-	-
*A*. *noctua*	+	+	-	-	-	-

The + Symbol indicates that serrations are present at the corresponding feather, the—Symbol indicates that serrations are missing. P: primary feather, gpc: greater primary covert.

We decided to investigate feathers from further bird species like *A*. *arvensis*, *C*. *livia domestica*, *L*. *fuscus*, *Lanius spec*., *P*. *strigoides*, and *S*. *habroptilus* for the description of a serration and to work out how serrated feather barbs deviate from non-serrated feather barbs (see S1 Table). *P*. *strigoides* and *S*. *habroptilus* were chosen, because Mascha [3] described serrations at the leading edge of these species. *A*. *arvensis*, *L*. *fuscus*, *Lanius spec*., and *C*. *livia domestica* served as reference species for non-serrated leading edges. The feathers of *A*. *arvensis*, *L*. *fuscus*, *Lanius spec*., *P*. *strigoides*, and *S*. *habroptilus* were investigated at ZFMK Bonn.

### Analysis of serrations

#### Data basis

Primary feathers and the leading edges of wings of *A*. *arvensis*, *C*. *livia domestica*, *L*. *fuscus*, *Lanius spec*., *P*. *strigoides*, *S*. *habroptilus* and of *T*. *furcata pratincola* served to describe serrated sections in comparison to non-serrated sections. The shape of serrations was determined on 10^th^ primaries of the 7 owl species selected. A total of five 10^th^ primaries were investigated per species.

#### Coordinate systems

**Fig 3:**
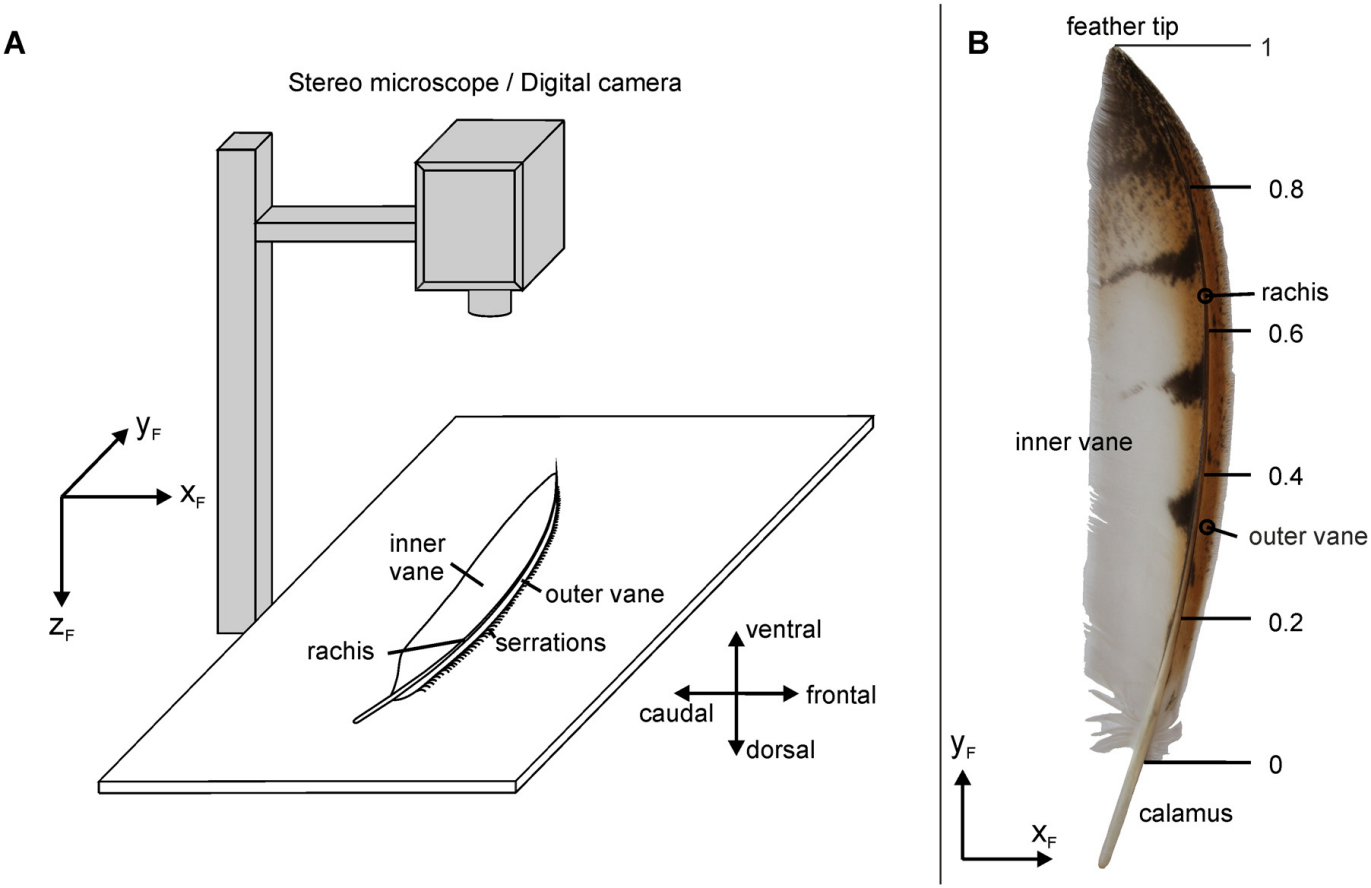
Measurement of shape parameters of serrations. (A) Measurement setup. The outer vane was mounted in the horizontal plane (x-y plane). The y-axis is in the direction of the unbent barb shaft. Serrations were photographed with a microscope camera or a digital camera from above as indicated by the positive z-axis pointing downwards. (B) Sampling points on a 10^th^ primary. The proximal end of the feather vane corresponds to sampling position 0, the wing tip, the most distal part of the feather vane, is set as sampling position 1. Sampling positions of 0.2, 0.4, 0.6 and 0.8 were investigated.

A first Cartesian coordinate system was used to describe the gross anatomy of a feather (see inset in Fig 3A). The y-axis was defined as the connection of the beginning of the calamus and the tip of the rachis. This x-axis was perpendicular to this axis in the plane formed by the feather vane and pointed towards the outer vane. The z-axis pointed upwards, if the feather was examined upside up. We call the corresponding coordinates x_F_, y_F_ and z_F_ (Fig 3A). Note that the coordinate system was left-handed for feathers and barbs from the right wing and right-handed for feathers and barbs from the left wing. Measurements were done at four sampling points along the normalized y-axis of the feather vane (0.2, 0.4, 0.6, 0.8, Fig 3B) where the beginning of the outer vane was set as 0 and the tip of the outer vane was set as 1.

A second Cartesian coordinate system (x_B_, y_B_ and z_B_) was defined for the description of the barbs (Fig 1C). A barb consists of an elongated shaft and hook and bow radiates (Fig 1C). The shaft originates at the rachis and ends free standing. Typically, barb and hook radiates originate from the barb shaft and form the feather vane. The barbules are also elongated structures that lie in a plane (see Fig 1D in [6]). If we position the origin of this coordinate system at the point where the barb shaft originates at the rachis, and have the extent of the shaft forming the positive direction of the y_B_-axis, then the barbules extend in the x_B_-y_B_-plane of the coordinate system at the base of the barb (Fig 1C). The serrations are formed by barb endings at the outer vane of the feather (Fig 1C). In serrated feather parts, the radiates are reduced in length at the distal part of the barb which causes the separation of adjacent barbs.

#### Measurement procedures

The outer vanes of the investigated feathers were placed in a horizontal position. The feathers were placed upside down, because the velvety surface of the dorsal part of the feather vane obstructed a clear view on the barbs and the emerging serrations. Pictures of the outer vanes were taken with a digital camera (Nikon D70 digital single-lens reflex camera, Nikon cooperation, Tokyo, Japan) or a microscope camera (Scope Tek DCM500, Hangzhou Scopetek Opto-Electric Co., Hangzhou, Zhejiang Province, P. R. China) fixed to a stereo microscope (Nikon SMT 10-A, Nikon cooperation, Tokyo Japan). The digital camera or the stereo microscope was positioned above the feather vane (Fig 3A). The camera images were adjusted horizontally to obtain a two-dimensional ventral image of the x-y-plane of the feather.

Morphometric data were obtained from the photographs with measuring tools of ImageJ (National Institutes of Health, Bethesda, MD, USA) and CorelDRAW (Corel Corporation, Ottawa, ON, Canada).

We used a confocal laser-scanning microscope (Leica TCS SP2; Leica Microsystems GmbH, Wetzlar, Germany) to digitize single barbs or an array of consecutive adjacent barbs on primary feathers of owls and other bird species. We did these measurements to investigate the differences between serrated and non-serrated feathers. Single barbs were fixed to a slide with polymer clay (Fimo classic; Staedtler Mars GmbH & Co. KG, Nuremberg, Germany) similar as described by Bachmann & Wagner [6]. Arrays of adjacent barbs were observed on feather vanes that were placed at the confocal scanning laser microscope. The single barbs or barb arrays were positioned with the upper surface of the feather pointing upwards. In some cases, for example for barbs of *C*. *livia domestica*, the leading edge was positioned upwards to obtain a better view from a frontal perspective. A diode laser with a wavelength of 405 nm was used to excite the auto-fluorescence of feather keratin. The light emission was measured from the dorsal to the ventral direction or from the frontal to the caudal direction, depending on the orientation of the barb. The voxel size was set at 2.93 μm * 2.93 μm * 5 μm or 2.93 μm * 2.93 μm * 2.9 μm. Both resolutions were suitable to obtain a three-dimensional reconstruction. Similar as in the investigation by Bachmann & Wagner [6] single barbs and barb arrays had to be recorded in several consecutive scans to obtain a complete reconstruction of the feather part investigated. The recorded images were exported as image stacks in TIFF format. The three-dimensional reconstruction was done with the software AMIRA 4.1.1 (Mercury Computer Systems GmbH, Berlin, Germany). The recorded image stacks of a barb or a barb array were aligned with AMIRA to obtain the complete reconstruction of the object.

#### Quantification of serration shape

**Fig 4:**
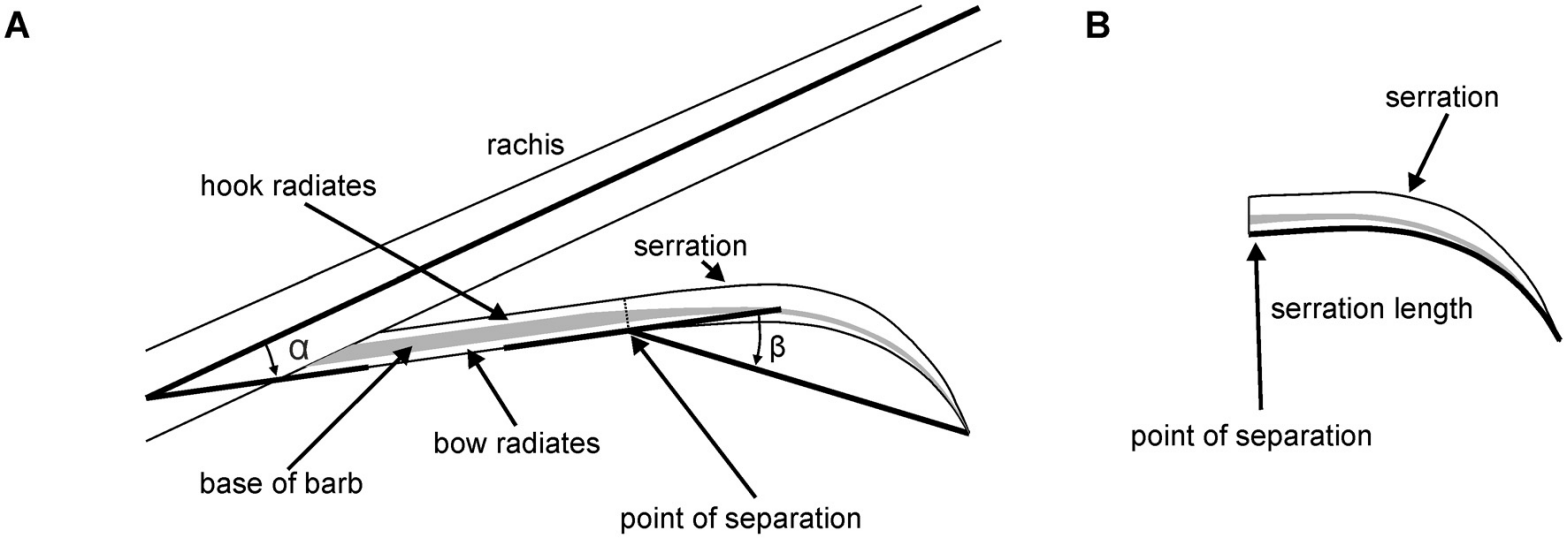
Quantification of barb and serration shape. (A) Rachis with a barb. The barb is divided into a base and a serrated tip. The base of the barb extends from the origin at the rachis to the point of separation. The positions of the bow and hook radiates are indicated as well. Two angles characterized the geometry. The inclination angle α is the angle between rachis and the base of the barb. The tip displacement angle β is the angle between the base of the barb and the tip of the serration measured from the point of separation. (B) Orientation and placement of a serration for the measurement of serration length.

The serrations are the endpoints of barbs. We characterized the shape of the serrations by three parameters. The first measure that we considered important in the quantification of the shape of the serrations is the inclination angle α, the angle between rachis and barb (Fig 4A). The barb first extends along a straight line. Bachmann and Wagner [6] called this part the base of the barb. Then the serration forms by the reduction of the bow and hook radiates leading to the separation of adjacent barbs. The point where this happens is called the point of separation in the following. After the serration is formed the barb shaft starts to bend. In a simple approach, the bending may be characterized by the angle between the base of the barb and the tip of serration as measured from the point of separation (Fig 4A), and this angle is called the tip-displacement angle β. The tip-displacement angle was our second characteristic. The length of a serration in the x_F_-y_F_-plane, the third parameter, was determined by using the Bezier Curve Tool of ImageJ. We determined the inclination angle, the tip displacement angle, and serration length for five serrations taken at each feather position in each feather.

#### Statistics

Statistical analysis was done in Matlab (The MathsWorks, Inc., Natick, MA, USA) and Excel (Microsoft Corporation, Redmond, WA, USA).

We used the Monte-Carlo method [14] to evaluate possible intraspecific and interspecific statistical differences of the inclination angle, tip displacement angle and serration length. The idea behind the Monte Carlo test was that the five data points obtained at each position were not independent of each other. The reason is that the serrations are densely packed and that, therefore, for example, the inclination angle does not vary independently from one serration to the next. Nevertheless, there was some variability in the measurements obtained at each position. To deal with the interdependence and to consider the variability we chose to randomly select one value at each position for each feather in the Monte Carlo tests and carried out a Mann-Whitney-U-test with two samples of five data points each. We used p<0.05 as statistical criterion. We ran this test 5000 times and counted how often the test exhibited a statistical difference. In this analysis zero percent means that none of the tests show a statistical difference and 100% means that all of the tests were significantly different at the p<0.05 level. We interpret that data such that 99% of significant runs indicates a difference at p<0.05, while a lower percentage of significant runs indicates a lower or no statistical difference.

We used the mean values of the parameters investigated for all seven owl species to create dendrograms. The mean values of one parameter were normalized to the highest value of this parameter. This sets the value of the species with the highest parameter at 1. These normalized values were the input to the MATLAB function linkage. Using this function, hierarchical binary cluster trees were created based on the unweighted average distance and using Euclidean metrics.

## Results

While it has long been known that owls have developed serrated structures at the leading edge of their wings, much less attention has been paid so far to the distribution and occurrence of serrations in owls of different sizes and owls having different activity patterns. We hypothesized that the occurrence, the distribution and the morphology of the serrations may depend on the size and the activity pattern of an owl species. In the following, we shall first describe how serrations may be characterized, then describe the occurrence of serrations over the wing and compare three shape parameters of serrations of the 10^th^ primary remex between owls of different sizes and activity patterns.

### Reinvestigation of the concept of a serration

Although many authors have mentioned the existence of serrations [1–7], no one has clearly worked out what a serration is. We ask here, how a serration may be defined and how the serrated part of a wing or feather may be distinguished from a non-serrated part.

**Fig 5:**
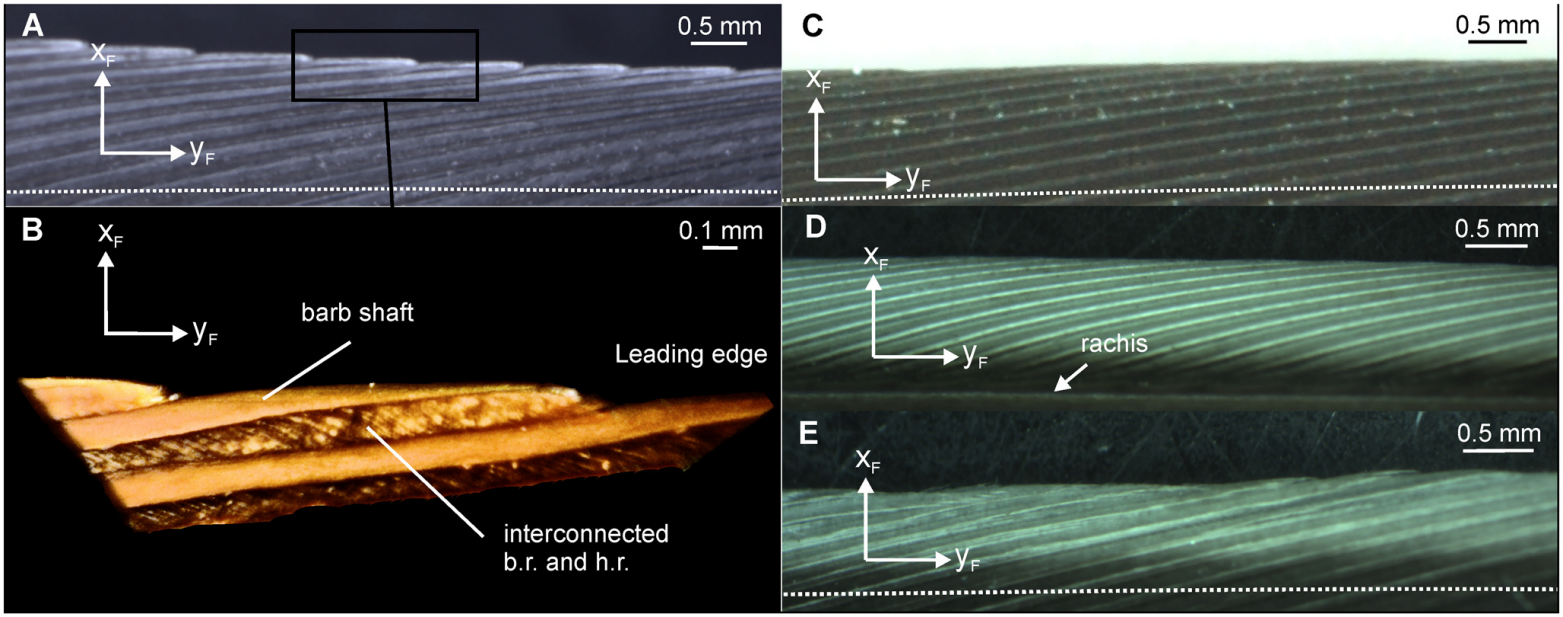
Three-dimensional reconstructions and images of outer vanes from different 10^th^ primaries. (A) Top view of a leading edge of *C*. *livia domestica*. The leading edge forms a distinct edge with small indentations at the interface between adjacent barbs. The white dotted line depicts the rachis of the feather in this area. Note the tilting of the barbs towards the rachis. (B) Three-dimensional reconstruction of consecutive barbs at the leading edge of *C*. *livia domestica*. The bow radiates are missing at the barb tip, exposing the outermost part of the barb. (C) Leading edge of 10th primary of *L*. *fuscus* (ventral view). (D) Leading edge of 10th primary of *A*. *arvensis* (top view). (E) Leading edge of 10th primary of *Lanius spec*. (top view). h.r.: hook radiates, b.r.: bow radiates.

A description of the leading edge of 10^th^ primary feathers that are not serrated served as reference. Such a non-serrated feather is the 10^th^ primary feather of *C*. *livia domestica*. The leading edge in this feather is more or less smooth with small indentations at the interface between adjacent barbs (Fig 5A). A three-dimensional reconstruction of adjacent barbs of the outer vane showed that the bow radiates are missing at the tip of the barb (Fig 5B). The bow radiates of a given barb disappear where the next, more proximally lying barb ends. By contrast the hook radiates are developed right up to the barb tip. The barb tips, formed by the barb shaft and the hook radiates, are attached to the most distal bow radiates of the adjoining barb. The outer edge of the vane that faces the air stream is mostly formed by a freestanding part of the barb shaft. The barb shafts are bent towards the rachis (Fig 5A).

The leading edges of 10^th^ primary feathers of *L*. *fuscus* (Fig 5C), *A*. *arvensis* (Fig 5D) and *Lanius spec*. (Fig 5E) look similar as that in *C*. *livia domestica*. Thus, the leading-edge feathers in most species have a more or less smooth leading edge with small indentations at the interface between adjacent barbs, and the barb shafts are straight or bent towards the rachis.

**Fig 6:**
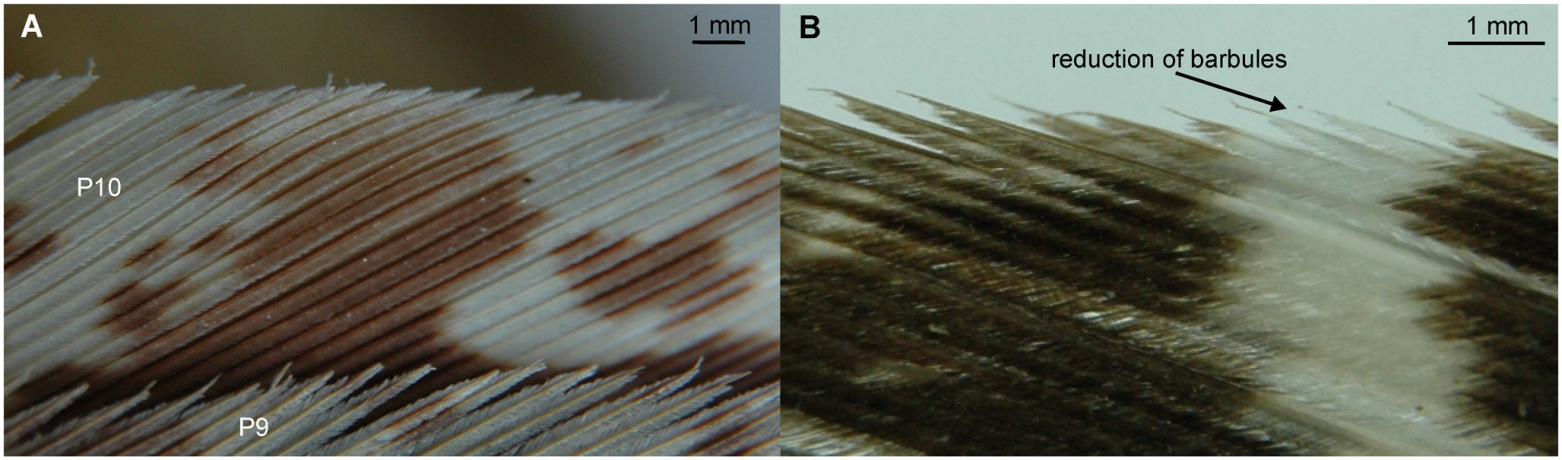
Leading edges on feathers of *S*. *habroptilus* and *P*. *strigoides*. (A) Horizontal view on flight feathers of *S*. *habroptilus* from a dorsal perspective. (B) Horizontal view on a 10^th^ primary feather of *P*. *strigoides* at 20% of the feather vane from a dorsal perspective. The barbules may be reduced in length at the barb tip.

The leading edges in some species are not smooth, but exhibit a saw-toothed or denticulate shape. For example, the barbs on leading-edge feathers of *S*. *habroptilus* (Fig 6A) and *P*. *strigoides* (Fig 6B) differ from the barbs shown in Fig 5. The tips of the barbs in these species are not attached to adjoining barbs but are free. The tips of the barbs protrude from the closed vane like spear heads giving the leading edge a denticulate shape (Fig 6). The exposure is further increased, in part, by a reduction in length of the barbules at the apical part compared with the length at the basal part of the barb (see arrow in Fig 6B). Similar to the barbs shown in Fig 5, the barb shafts are straight or bent towards the rachis (Fig 6A and 6B).

**Fig 7:**
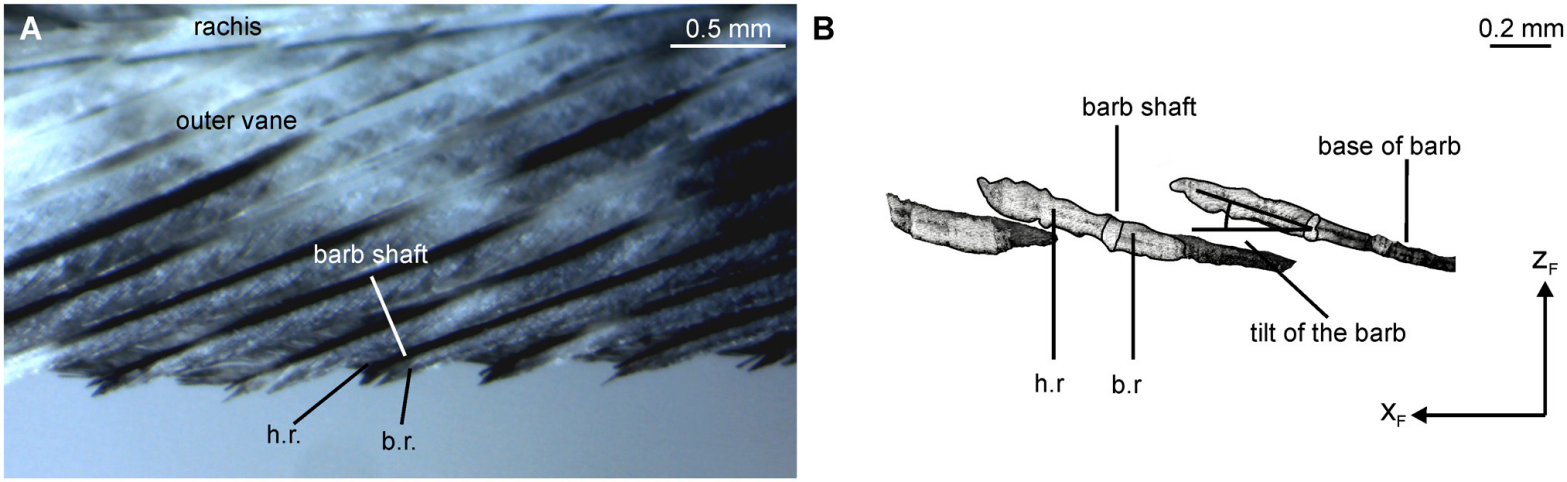
Outer vane of the 10^th^ primary feather tip of *T*. *furcata pratincola*. (A) Photograph of the leading edge of a 10^th^ primary of *T*. *furcata pratincola* taken at 0.95–0.97 of the vane length. Note the denticulate leading edge. Note also that the barb shafts are straight or bent towards the rachis. (B) Frontal view of a three dimensional reconstruction of the outer vane. The tips of three barbs are presented in this reconstruction. Note that the Y_f_-Z_f_ plane is perpendicular to the view present here, and can, therefore, not be seen. Note the tilt of the barbs. h.r.: hook radiates, b.r.: bow radiates

A photograph from a 10^th^ primary of *T*. *furcata pratincola*, taken at position 0.95–0.97, i.e. very close to the tip of the feather vane (Fig 7A), shows denticulate barb tips that have a similar shape as the barb tips in *S*. *habroptilus* and *P*. *strigoides* (compare Fig 6 with Fig 7A). The barb shafts are slightly bent towards the rachis or not bent at all, again similar to the situation shown in *S*. *habroptilus* and *P*. *strigoides* (Fig 6). Both, bow and hook radiates are present to the end of the barb shaft. Consequently, and in contrast to the situation shown in Fig 5B, the outer edge of the vane does not appear smooth, but denticulate as those shown in Fig 6. The tip of the barb shaft ends abruptly, as if broken. It is not freestanding, but also not covered by barbules (Fig 7A). The plane formed by a barb shaft and its barbules is tilted upwards in the x_F_-z_F_ plane (Fig 7B), an orientation which we also observed at outer vanes of *C*. *livia domestica*.

Although the leading edges in the example shown in Figs 6 and 7 are less smooth than those shown in Fig 5, we do not consider these feathers or these parts of a feather as serrated. Thus, a detachment of barb tips leading to the denticulate leading edge is in our opinion not sufficient to speak of serrations.

**Fig 8:**
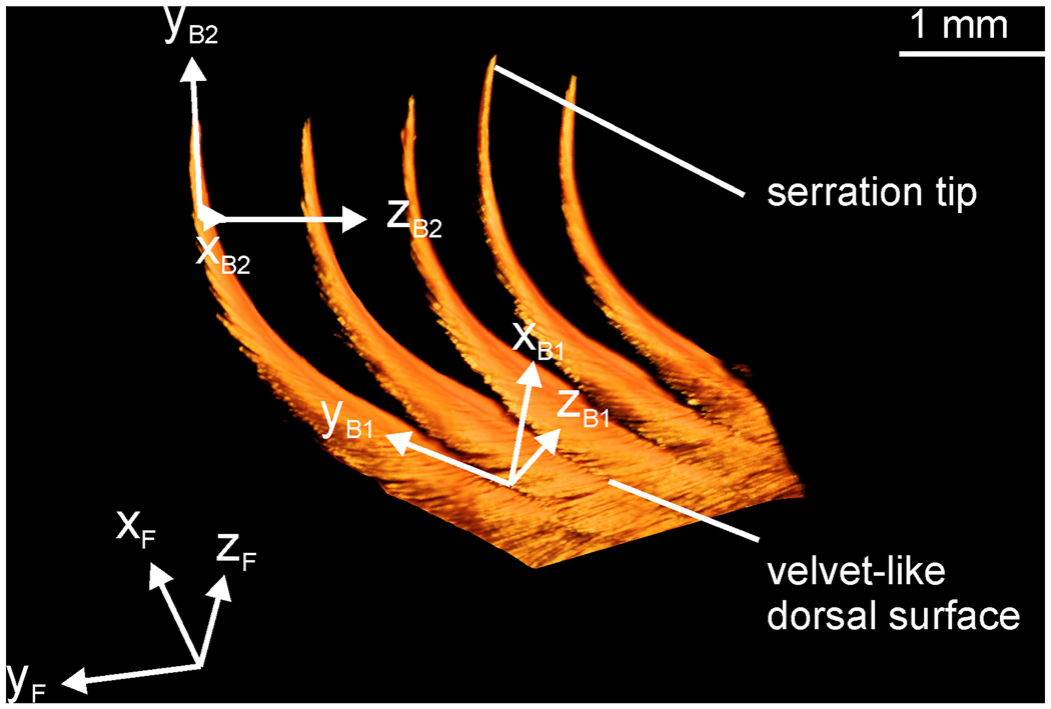
Serrations at 50% of the outer vane on a 10^th^ primary feather of *T*. *furcata pratincola*. Isometric-like view on a three-dimensional reconstruction of five serrations. Note the upward bending and the twisting as indicated by the coordinate systems (for more information see text).

**Fig 9:**
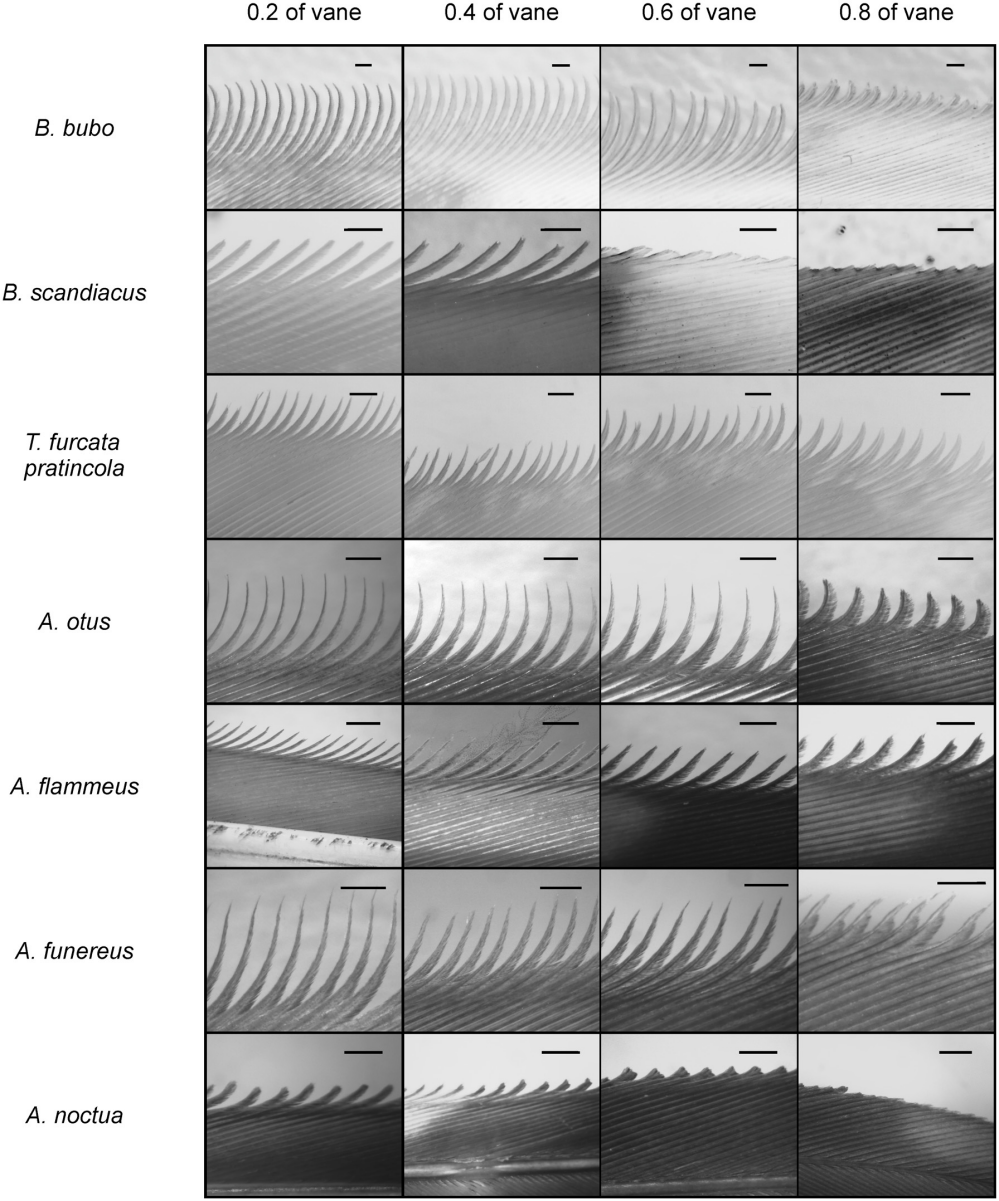
Serrated structures on owl wings. The serrations of all 7 investigated owl species are presented for 4 different measurement positions. Note the variation in shape between the different species and between the different positions within one species. The black bars represent 1 mm.

At more central parts of a 10^th^ primary feather of *T*. *furcata pratincola* the orientation of the barbs starts to differ from the orientations described before (Figs 8 and 9 third row from the top). The barbs are no longer straight or bent towards the rachis, but are bent in the opposite direction, away from the rachis. This bending was not present in the barb arrangements of the feathers examined above. A closer look at these barbs reveals that the bending has several components: apart from bending away from the rachis, the apical part of the barbs also bends upwards and thereby twists (Fig 8). The twisting is best seen by the orientation of the plane formed by the barb shaft and its attached barbules, the x_B_-y_B_ plane. At the base of the barb this plane, called x_B1_-y_B1_ plane, is close to the x_F_-y_F_ plane. The x_B1_-y_B1_ plane is slight tilted upwards with respect to the y_F_-z_F_ plane as was already shown in Fig 7A for a different part of the vane. By contrast, at the tip of the serration this plane, now called x_B2_-y_B2_ plane, has rotated by about 90 degrees so that the x_B2_ axis faces the viewer (Fig 8).

In summary, we suggest that a serration does not only require a detached barb tip, but also a bending of the tip away from the rachis as defining characteristics. The bending is augmented by upward bending and twisting. Applying this definition, we observed serrations only on owl feathers.

### Occurrence of serrations

We found leading-edge serrations on different wing feathers including remiges, alulae and coverts. The different owl species exhibited different serrated feathers (see Table 1). In the large, nocturnal *B*. *bubo* serrations were observed on the 7^th^ to 10^th^ primary feathers, the 3rd Alula, and on the 10th greater primary covert (Figs 2 and 9 upper row, Table 1). By contrast, in the diurnal *B*. *scandiacus*, with a similar size as *B*. *bubo*, serrations only occurred at 10^th^ primaries (Fig 9, second row from above). This was similar to the middle-sized *T*. *furcata pratincola* (Fig 9, third row from above). *T*. *furcata pratincola* also exhibited smaller serrations on the 10^th^ greater primary covert. The other two middle-sized species, *A*. *otus* (Fig 9 fourth row from the top) and *A*. *flammeus* (Fig 9 fifth row from the top) had serrations at the 9^th^ and 10^th^ primaries, despite their different activity patterns. In the small owls, serrations occurred at the 8^th^ to 10^th^ primaries in the nocturnal *A*. *funereus* (Fig 9 6^th^ row from the top), while in the diurnal *A*. *noctua* only the 9^th^ and 10^th^ primaries carried serrations (Fig 9, bottom row).

An examination of the 10^th^ primaries in the seven species showed that serrations were well developed at positions 0.2 and 0.4 of the feather vane, while the serrations at position 0.8 of the feather vane were reduced in length in all species (Fig 9). At position 0.6 of the feather vane the serrations in *B*. *scandiacus* and *A*. *noctua* were also reduced in size (Fig 9). We classified the serrations at position 0.6 in *B*. *scandiacus* and in *A*. *noctua* as “Surnia type”. The buds seen at the tips of the outer vanes at position 0.8 of the feather vanes in these two species did not fulfill our definition of a serration (Fig 9).

Since in our view there is a smooth transition from the “Surnia type” to the “Bubo type”, and since we could not identify a unique criterion for a classification, we refrain from a further quantification of the range over which the “Bubo type” of serration extends along the primary leading edge of the wing. At the positions 0.2 and 0.4, but not at the positions 0.6 and 0.8, along the feather vane, “Bubo type” of serrations were observed in all owl species examined (Fig 9). We use the variations occurring at these two positions to compare the serrations across species and across activity patterns.

### Morphological characteristics of Bubo-type serrations

For the following analyses, serrations occurring at sampling points 0.2 and 0.4 of the vane of the 10^th^ primaries of all owl species were compared. As mentioned in Materials and Methods, three parameters were chosen for the comparison. Before we show the data we mention that a within-species comparison of serration shape at these two positions showed that the serrations at these two positions were very similar in each species for each of the parameters tested (Mann-Withney U test).

### The inclination angle

**Fig 10:**
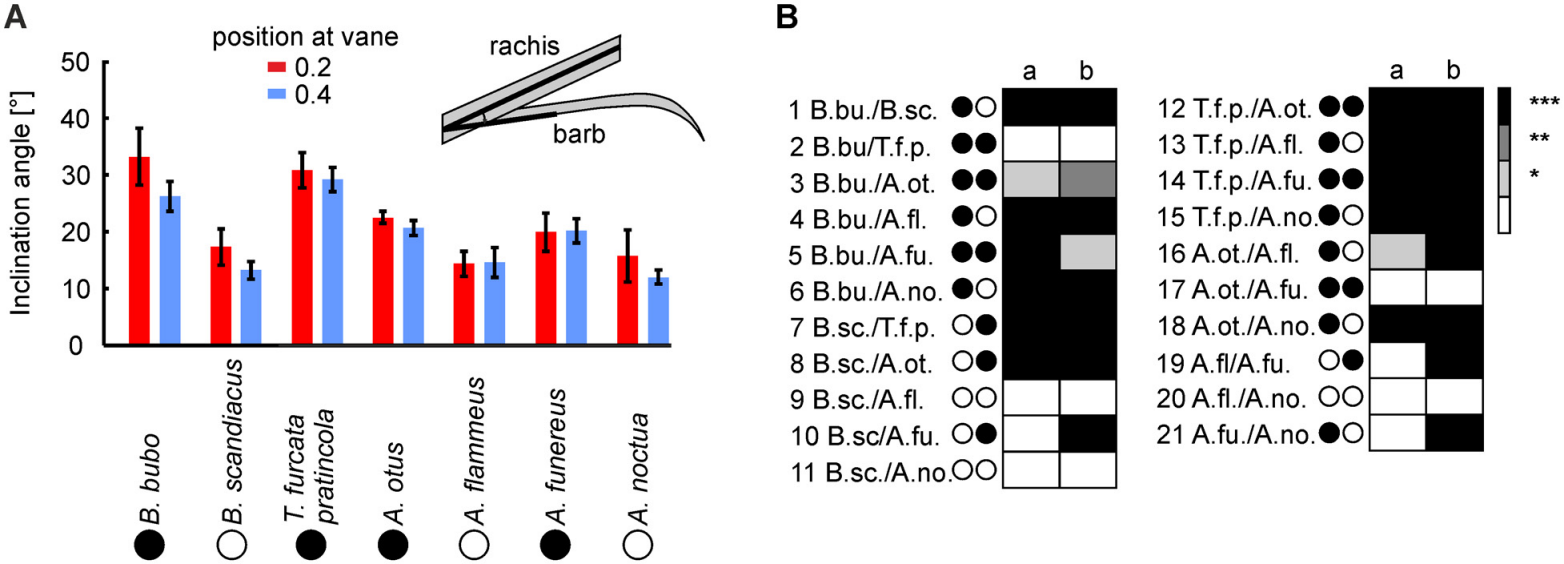
Inclination angles. At each position, the inclination angles of five serrations were measured for each feather. Five feathers were measured per species and feather position resulting in 25 measurements per species and feather position. Black circle: nocturnal species, white circle: diurnal species. (A) Plotted are the mean values and standard deviations of these 25 measurements. The inset in (A) shows how the inclination angle was measured. The color of the bars depicts the position at the feather as indicated in the inset. (B) Statistical comparison. Results of comparisons between species and positions. Abbreviations: B. bu.: *B*. *bubo*; B. sc.: *B*. *scandiacus*; T.f.p.: *T*. *furcata pratincola*; A. ot.: *A*. *otus*; A. fl.: *A*. *flammeus*; A. fu.: *A*. *funereus*; A.no.: *A*. *noctua*. *** and black: significance in more than 99% of the runs; ** and dark grey: significance in more than 95% of the runs, but less than 99% of the runs; * and light grey: significance in more than 67% of the runs, but less than 95% of the runs; white: significance in less than 67% of the runs. Note that for different activity patterns in 20 of 24 comparisons 99% of the runs yielded significance.

The leading-edge serrations emerge from the barbs of the outer vane that offset from the rachis at a distinct angle, the inclination angle (Fig 4A, see also inset in Fig 10A). The inclination angle is our first criterion in the quantification of serration morphology. The mean inclination angles for the diurnal species ranged from 12° in *A*. *noctua* at 0.4 of vane length to 17.3° in *B*. *scandiacus* at 0.2 of the feather vane (Fig 10). In comparison, the mean inclination angles for nocturnal species were all above 17.5°, the minimal angle being 19.9° at 0.2 of the feather vane in *A*. *funereus*. The largest angle, at 0.2 of the feather vane in *B*. *bubo*, was at 33° (Fig 10A).

Since the inclination angles of the densely packed barbs are not independent of each other, we used the Monte-Carlo method as described in Materials and Methods to find out whether the observed variations were statistically different. This method suggested that the inclination angles in the diurnal species were not different from each other. In all 6 comparisons were the runs significant in less than 67% of the cases (Fig 10B). Thus, in diurnal species the inclination angle did not depend on size. The 12 comparisons between the nocturnal species yielded a mixed picture: 3 of 10 comparisons exhibited differences in more than 99% of the cases, while the other 7 comparisons did not (Fig 10B). Differences were also seen when the nocturnal and diurnal birds were compared: in 15 of 16 comparison a significant difference was observed in more than 99% of the runs, if activity patterns and size were taken into account (Fig 10B). This difference was mainly based on activity pattern, because also in 20 of 24 comparisons using only activity pattern as criterion, 99% of the runs yielded significance. This suggested to us that birds of different activity patterns tend to have different inclination angles with the inclination angles being higher in nocturnal than in diurnal species. By contrast, size had a less distinct influence on the inclination angle than activity pattern.

### Tip-displacement angle

**Fig 11:**
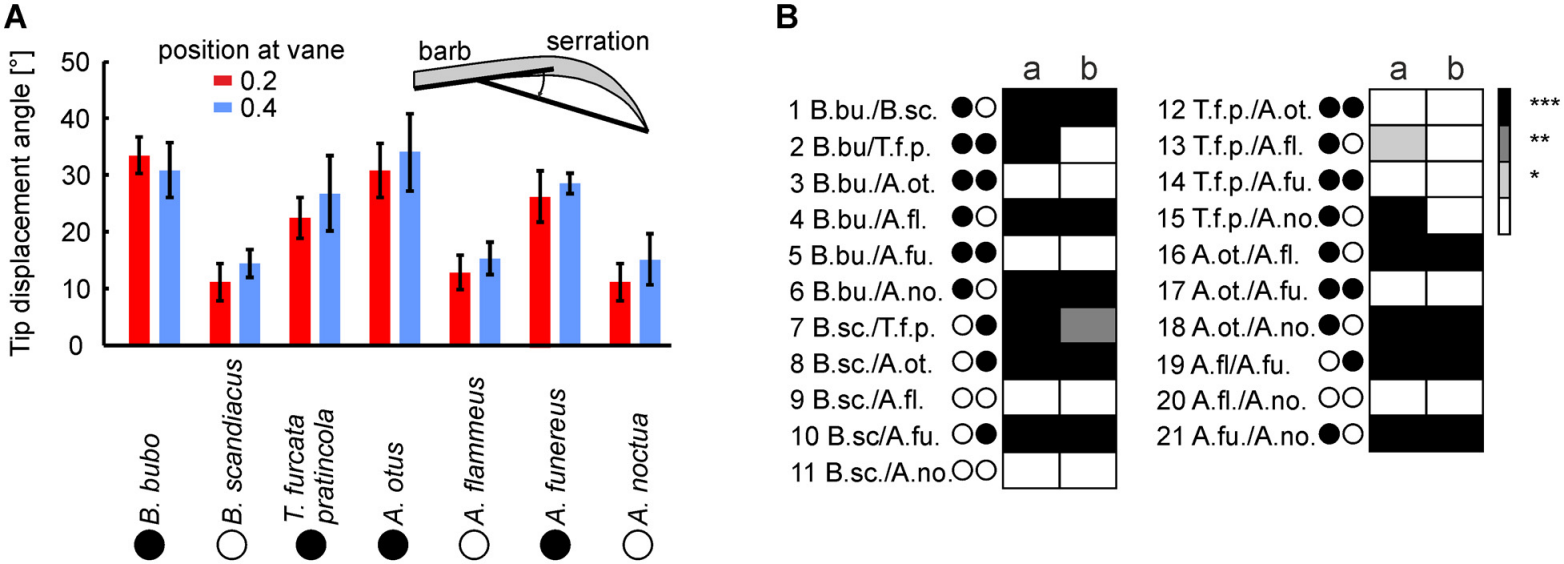
Tip displacement angles. (A) Plotted are the mean values and standard deviations of these 25 measurements. The inset in (A) shows how the displacement angle was measured. The color of the bars depicts the position at the feather. (B) Statistical comparison. The data base is the same as in Fig 10, and the measurement results for the tip displacement angle of all investigated owl species were compared in the same way as the inclination angles (see legend to Fig 10). Note that similar to the data for the inclination angle, in 20 out of 24 comparisons, more than 99% of the runs were different.

At the apical part of the barb, the serration takes a curved form (Figs 1 and 4) and bends away from the rachis. The tip-displacement angle was evaluated as a simple indicator of the bending. The tip-displacement angle is the angle between the orientation of the base of the barb and a line from the point of separation to the tip of the serration (Fig 4A, see also inset in Fig 11A). The tip displacement angles ranged from about 10 degrees to more than 30 degrees (Fig 11A). The tip displacement angle did not depend on size in both the diurnal and nocturnal species. In 6 of 6 and 9 of 10 comparisons between diurnal and nocturnal species of different sizes, respectively, less than 67% of the runs yielded significance (Fig 11B). By contrast, more than 99% of the runs yielded significant differences in 14 of 16 cases, if tip-displacement angles were compared for the species at equivalent positions on the wing having different activity patterns and different sizes. As with the inclination angle, this difference was mainly due to the different activity patterns (in 20 out of 24 comparisons more than 99% of the runs were different). This suggested that the tip-displacement angle at equivalent positions on the wing is at least 47% larger in nocturnal species than in diurnal species (Fig 11A).

### Length of serration

**Fig 12:**
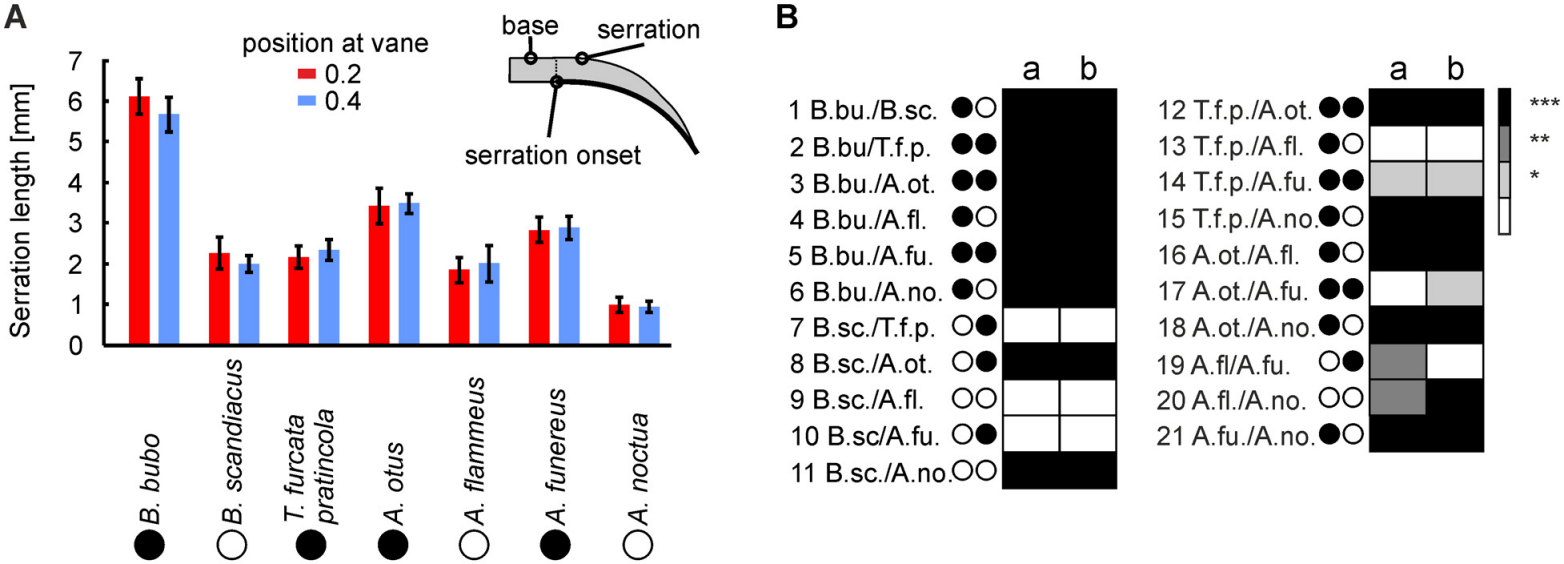
Lengths of the serrations. (A) Plotted are the mean values and standard deviations of these 25 measurements. The inset in (A) shows how the length of the serrations was measured. The color of the bars depicts the position at the feather. (B) Statistical comparison. The data base is the same as in Fig 10, and the measurement results for the serration length of all investigated owl species were compared using the Monte Carlo method in the same way as the inclination angles (see legend to Fig 10). Note that serration length seems to be determined by a mixture of size and activity pattern, with no clear separation according to either of the two.

The length of a serration in the horizontal or x-y plane (Fig 4B, see inset in Fig 12A) was used as the third criterion in the characterization of the morphology of the serrations. The length of a serration was determined from the point of separation to the tip along the curved path. The length of a serration depended on the species investigated (Fig 12A). The longest serrations could be found in *B*. *bubo* with a length between 5 and 7 mm (Fig 12A). These serrations were significantly longer than serrations of all other investigated species (Fig 12B). *A*. *noctua* exhibited the shortest serrations of all species investigated with a length of about 1 mm. There was a tendency of the increase of serration length with size of a species. In 3 out of 6 comparisons and 6 out of 10 comparisons between the diurnal and nocturnal species, respectively, a difference was observed in more than 99% of the Monte Carlo runs (Fig 12B). This held also when both size and activity pattern were taken into account. Now a difference was seen in 10 out of 16 comparisons in more than 99% of the runs. If activity pattern was compared independent of size, 16 out of 24 comparisons exhibited a difference in more than 99% of the runs (Fig 12B). As mentioned before, the serrations of *B*. *bubo* were longer than the serrations of all other species investigated (Fig 12A and 12B), while the serrations in *A*. *noctua* were shorter than the serrations in the all other species investigated. The serrations of *T*. *furcata pratincola* from the family *Tytonidae* were significantly shorter than serrations of the same sized *A*. *otus* and the larger *B*. *bubo*, and tended to be also shorter than those of *A*. *funereus* who all belong to the *Strigidae* family (Fig 12B). The length of the serrations of *T*. *furcata pratincola* was not different from that of *B*. *scandiacus* and *A*. *flammeus*. Thus, serration length seems to be determined by a mixture of size and activity pattern, with no clear separation according to either of the two.

### Dendrograms

**Fig 13:**
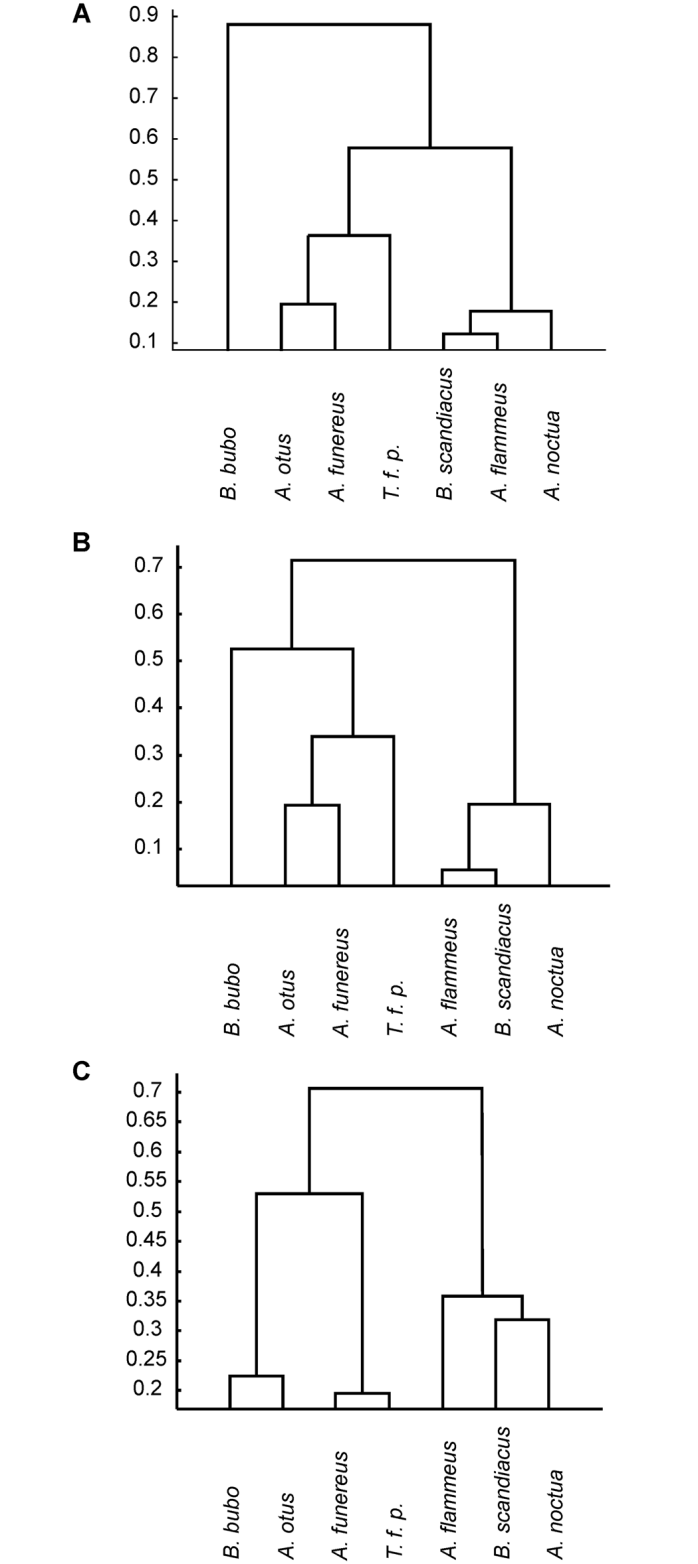
Dendrogram. Dendrograms were separately created for the different measurement positions based on the unweighted average distance and using the Euclidean distance metric at the corresponding position. The values next to the branches represent the distance between data points. (A): 0.2 of vane. (B): 0.4 of vane. (C) 0.6 of vane. Note that in (B) and (C) the diurnal species are clustered separately from the diurnal species, while in (A) the biggest species, *B*. *bubo* is clustered separately, while the remaining 6 species are clustered according to their activity pattern.

We used the mean values as plotted in Figs 10A, 11A and 12A to calculate dendrograms. The situation at positions 0.2, 0.4, and 0.6 were investigated separately. Note that we chose here to also include data at position 0.6 for a comparison, although some of the serrations were not classified as Bubo type. The dendrogram resulting for position 0.2 separated *B*. *bubo* from the other owl species (Fig 13A). Within the 6 remaining species the dendrogram separated between nocturnal and diurnal species. We interpret this result as showing an influence of size, because *B*. *bubo* is the by far the largest owl species, but also an influence of activity pattern, because all diurnal species were clustered separately from the nocturnal species. At position 0.4 of the feather vane, the clustering algorithm completely separated the nocturnal from the diurnal species (Fig 13B). This suggested an influence of activity pattern only on the development of the serrations at this position. An equivalent dendrogram as for position 0.4 resulted at position 0.6 (Fig 13C, measured data not shown).

## Discussion

We observed leading-edge serrations within the order of owls (*Strigiformes*), but not in other bird species examined. The analysis of three selected parameters demonstrated that serration shape depends on the activity pattern of a species and, to a lesser extent, on species size. In the following we first discuss what we consider a serration, then set our findings on the shape of serrations in context with the existing literature, and finally attempt to draw conclusions about serration function.

### Occurrence and shape of serrations

Despite a description of serrations in several earlier studies [1,3–7], a clear definition for a serration was lacking so far. The problem was not so much to discriminate smooth leading edges from serrated leading edges, but to discriminate between denticulate leading edges (Figs 6 and 7) and serrated leading edges (Figs 8 and 9). Mascha [3] had suggested that the denticulate leading edges of *P*. *strigoides* and *S*. *habroptilus* should be considered to be serrated. These denticulate barbs are straight or bent towards the rachis as do barbs from the leading edge in pigeons and other “non-specialized” species. By contrast, the serrations in owls are bent away from the rachis. Since this bending away from the rachis is found in all serrated regions, we suggest using this bending as discriminating criterion between denticulations and serrations. We found that the bending and orientation of the barbs was by far the best defining characteristic of a serration. When we tried to use inclination angles and serration lengths as defining characteristics, we obtained ambiguous classifications. Inclination angles also depended on the general feather morphology. Serration length was very variable. For the discrimination of serrated and non-serrated, specifically denticulate parts and short serration lengths would be interesting. These proved to be too variable as well.

Bachmann and Wagner [6] had already stated that serrations on the wings of *T*. *furcata pratincola* were oriented away from the rachis, but had not studied other species. Using this definition, some regions of the leading edges of owls, specifically at the most distal parts of the outer vane of 10^th^ primaries are also non-serrated, but have denticulations or indentations. We were concerned that the free-standing denticulate tips in some owl feathers might be due to damage at the tip, and were cautious to select specimens where the feathers were neither damaged by mechanical wearing nor by microbial degradation or parasite infestation. We did not observe damage of the barbs that could explain the change of bending direction.

Our observations support Sick’s [4] suggestion that serrations have different shapes. While we observed serrations that corresponded to Sick’s [4] classification, the “Bubo-type” and the “Surnia-type” serrations, we also observed a wide range of shapes and sizes. Thus, as Sick [4] had already indicated, we came to the conclusion that a classification into two categories may be too simple. Although we saw these two types, we also observed many intermediate shapes, and we could not find an abrupt transition. Furthermore, we found serrations on the wings of all owls we investigated that were of the “Bubo type”. These serrations were typically found at middle positions along the feather vane of 10^th^ primaries. By contrast, at the distal part of all owl species investigated “Surnia-type” serrations occurred. These variations suggested that different types of serrations may occur within one species. This, argues against a restriction of one type of serrations to one species. Since the transition from one type to the other was smooth, we refrained from suggesting a regionalization of the wing into a “Bubo type” and “Surnia type” regions.

### Comparison of the intra- and interspecific characteristics of serrations

Serrations are not uniformly shaped but show a large variety between different species as well as different feather positions [1,3,6,7,15]. These differences in serration shape can especially be seen between nocturnal owls that rely on a silent flight and tend to fly slowly, and diurnal or insectivore species that may hunt at higher flight velocities. The differences in serration shape were obvious by serration size and orientation. These differences were present even for similar-sized species of the same genus. This suggested to us that, even if the body size correlates to the taxonomy for the investigated Strigidae species, the shape of the serrations, especially the orientation has developed in regard to the activity of the owl species.

The serrations of nocturnal species were more aligned with the airflow than the serrations of diurnal species, indicated by the larger inclination angles and tip-displacement angles in the nocturnal species. Note that the sum of the inclination angle and the tip-displacement angle in no case exceeded 90°. A sum of the two angles of 90 degrees would mean that the serration tip is directly facing against the airstream, if we assume that the rachis of the 10^th^ primary is perpendicular to the air stream. Our data are consistent with those of Bachmann & Wagner [6]. Note, however, that this reasoning only holds for gliding flight, when the wing is not moving relative to the body, but not during flapping flight [16–19]. Note also that the tip displacement angle may underestimate the direction of the tip. The differences in alignment of the serrations goes along with the serrations of diurnal species being shorter than the serrations of nocturnal species of similar size. Furthermore, the serration length of the investigated species was related to body size. This held specifically for *B*. *bubo* that has by far the longest serrations. A conspicuous exception was the serration size of *T*. *furcata pratincola* who were significantly shorter than the serrations of the similar sized *A*. *otus* and had a similar length than the serrations of the smaller *A*. *funereus*. These comparatively small serrations of *T*. *furcata pratincola* might be influenced by phylogeny, because this species is the only investigated specimen of the family *Tytonidae*. Different serration sizes for different species have been described previously [1,6,7,15]. It is tempting to speculate that the serrations length is adapted to aerodynamic properties that depend on wing size (see also next section). More data are necessary, however, including also the analysis of the twisting of the serrations, to obtain a clearer picture on this issue. Since we wanted to compare serrations in different species here, this was not the focus of this study.

### Possible functions of serrations

The silent owl flight has been associated with wing and feather adaptions in this order of birds. Owls exhibit large wings in comparison to their body weight that enable a flight at slower velocities [20], a crucial advantage, since flight noise emission is dependent on the flight velocity [21,22]. Recent comparisons of flight in owls species with flight in diurnal predators support the theory that feather adaptations in owls indeed have a noise reducing effect even at similar flow velocities [7,23–25]. The leading-edge serrations are supposed to change the air flow so that noise emission is reduced during flight [5,26]. Wind tunnel studies on model wings also demonstrated that serrations attached to artificial airfoils influenced the air flow around the wing [27–33]. Serrations effect the laminar-turbulent transition [27] and stabilize the flow over a large range of velocities and different angles of attack [28]. Other experiments demonstrated noise reduction on airfoils equipped with serrations in comparison to non-serrated airfoils [29,30]. Neuhaus et al. [5] compared noise generation of wings of the tawny owl (*Strix aluco*) with serrations and with serrations removed. These authors concluded that serrations might be important during high angles of attack, for example during critical flight maneuvers.

If the natural serrations have indeed a beneficial effect on the reduction of flight noise of owls, they should be particularly important for nocturnal owl species that rely on such a silent flight. The more developed shape of the serrations at 10^th^ primary remiges of nocturnal species in comparison to serrations of diurnal species that we observed in this study supports this claim. A recent study by Winzen et al. [28] reported that artificial serrations attached to an owl-wing model caused a stabilization of the air flow at a Reynold’s numbers in the range of the barn-owl flight, with the drawback of a reduced aerodynamic performance due to a decreased lift-to-drag-ratio. If similar effects were caused by natural serrations as well, the existence of serrations in owls that do not rely on a slow, silent flight would be detrimental for their aerodynamic performance. This disadvantage may be the reason why serrations are less well developed in diurnal owls and are missing in all other bird species. This brings up the question why diurnal owl would have serrations at all? The answer might lie in phylogeny. The phylogenetic tree of owls [13] suggests that serrations are a plesiomorphic characteristic for the Strigiformes. More data are needed, however, on the development and function of serrations. It is, for example, not yet clear to what extent the studies on wing models can be transferred to real owl wings, because Winzen et al. [34] described aerodynamic differences between natural and model owl wings.

## Conclusions & Outlook

We compared the serrations of the primary leading edge on wings from different owl species. In general we primarily selected our species based on the size and activity of the species with a regard to phylogenetical relationships. Other aspects like influences of the habitat to the development of feather adaptations were not considered for the selection. A detailed two-dimensional analysis of the serrations provided quantitative information about species-specific differences. The differences of serration shape between the diurnal and nocturnal owl species that we observed suggested that the serrations have indeed an important function for silent flight. However, only studies in acoustic wind tunnels with serrated and non-serrated real wings can show the effect of the serrations on the noise produced by a wing during flight.

Our study and most previous studies on owl wings focused on the primary leading edge where serrations on the 10^th^ primary remex were examined. Owls of the *Strigidae* family, like several other groups of birds, have developed slotted wings and resulting secondary leading edges [35]. These secondary leading edges are supposed to reduce the induced drag at the tip region of bird wings [36–38]. The serrations occurring on secondary leading edges have been little investigated in regard to their shape and functionality. Further analysis of such wings and serrations is necessary for a better understanding of the reduction of noise and airflow control by serrations. Such data can then be made available in a biomimetic sense for the construction of technical airfoils or ventilator blades.

## Supporting Information

S1 Fig Simplified phylogram of investigated owl species. The phylogram describes the phylogenetic relationship of all owl species that were investigated in this study. The phylogram is a simplified version of the ML bootstrap phylogram from [13]. This phylogram indicates that similar sized owl species within the Strigidae family that were used in this study belong within the same genus (Bubo, Bubonini and Asio, Asionini) or the same subfamily (Surniinae). An exception is the medium sized *T*. *furcata pratincola* which belongs to the outgroup of Tytonidae.
(TIF)

S1 Table Overview of bird species investigated. The species are sorted alphabetically. The weight and body size estimations include male and female animals, which can result in larger size differences. Information about the owl species is taken from [9]. Information about other bird species was taken from the handbook of the birds of the world (Del Hoyo J, Elliott A, Sargatal J, Christie DA (from 1997) Handbook of the birds of the world. Vol 1–16. Lynx Editions, Barcelona).
(DOCX)
