# Supplementary figures and images for: Morphological Variations of Leading-Edge Serrations in Owls (Strigiformes)
Source: PLoS One. 2016 Mar 2;11(3):e0149236. doi: 10.1371/journal.pone.0149236 (PMC4774958; doi:10.1371/journal.pone.0149236)

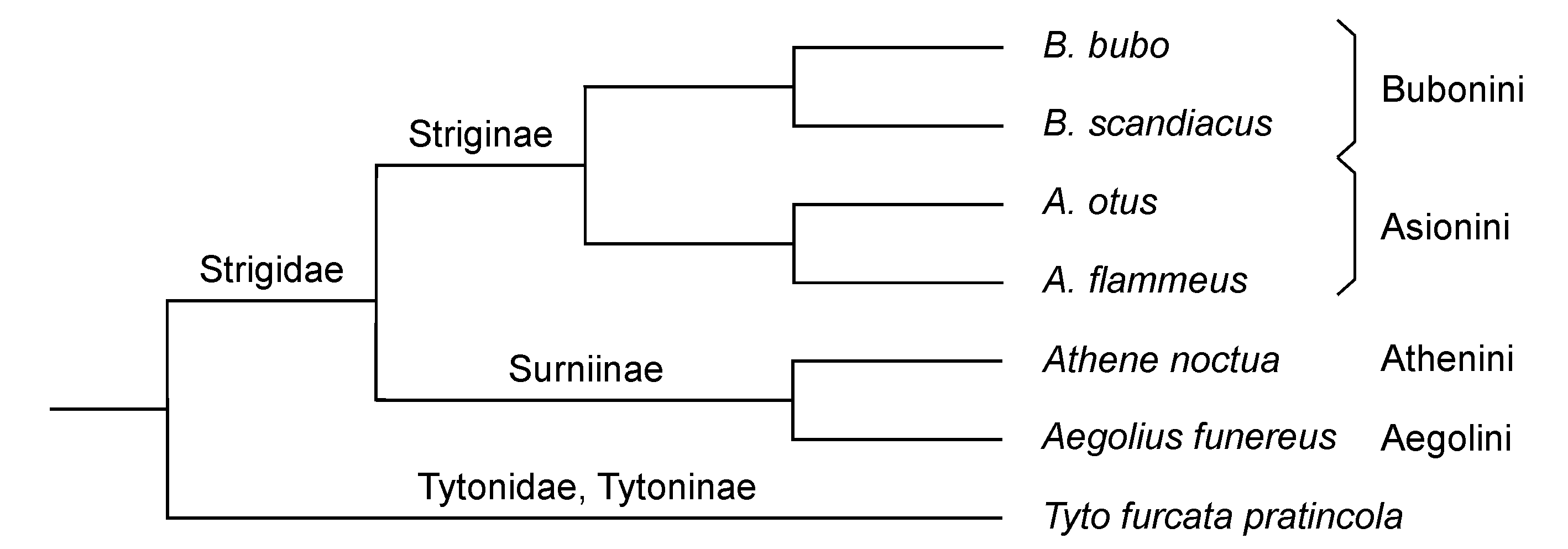

Supplement: S1 Fig — The phylogram describes the phylogenetic relationship of all owl species that were investigated in this study. The phylogram is a simplified version of the ML bootstrap phylogram from [13]. This phylogram indicates that similar sized owl species within the Strigidae family that were used in this study belong within the same genus (Bubo, Bubonini and Asio, Asionini) or the same subfamily (Surniinae). An exception is the medium sized T. furcata pratincola which belongs to the outgroup of Tytonidae. (TIF) [file pone.0149236.s001.tif]
