# Supplementary material for: Morphological Variations of Leading-Edge Serrations in Owls (Strigiformes)
Source: PLoS One. 2016 Mar 2;11(3):e0149236. doi: 10.1371/journal.pone.0149236 (PMC4774958; doi:10.1371/journal.pone.0149236)
Supplement: S1 Table — The species are sorted alphabetically. The weight and body size estimations include male and female animals, which can result in larger size differences. Information about the owl species is taken from [9]. Information about other bird species was taken from the handbook of the birds of the world (Del Hoyo J, Elliott A, Sargatal J, Christie DA (from 1997) Handbook of the birds of the world. Vol 1–16. Lynx Editions, Barcelona). (DOCX) [file pone.0149236.s002.docx]

S1 Table Overview of bird species investigated.

| **Species** | **Family** | **Weight** | **Body Size (total length)** | **Activity** |
| --- | --- | --- | --- | --- |
| *Aegolius funereus* | Strigidae | 90-194 g | 23-26 cm | nocturnal |
| *Alauda arvensis* | Alaudidae | 26-50 g | 16- 19 cm | diurnal |
| *Asio flammeus* | Strigidae | 206-475 g | 34-42 cm | diurnal |
| *Asio otus* | Strigidae | 210-430 g | 35-40 cm | nocturnal |
| *Athene noctua* | Strigidae | 105-260 g | 21-23 cm | diurnal |
| *Bubo bubo* | Strigidae | 1550-4200 g | 58-71 cm | nocturnal |
| *Bubo scandiacus* | Strigidae | 710-2950 g | 53-66 cm | diurnal |
| *Columba livia domestica* | Columbidae | 180-355 g | 31-34 cm | diurnal |
| *Lanius spec.* | Laniidae | < 100 g | <31 cm | diurnal |
| *Larus fuscus* | Laridae | 550-1200 g | 51-61 cm | diurnal |
| *Podargus strigoides* | Podargidae | 205-364 g | 34-53 cm | nocturnal |
| *Strigops habroptilus* | Strigopidae | 950-3000 g | 64 cm | nocturnal |
| *Tyto furcata pratincola* | Tytonidae | 290-560 g | 38 cm | nocturnal |
